# Supplementary material for: Arbuscular Mycorrhizal Fungi and Rhizobium Improve Nutrient Uptake and Microbial Diversity Relative to Dryland Site-Specific Soil Conditions
Source: Microorganisms. 2024 Mar 27;12(4):667. doi: 10.3390/microorganisms12040667 (PMC11052256; doi:10.3390/microorganisms12040667)
Supplement: Supplementary file 1 [file microorganisms-12-00667-s001.zip › microorganisms-2931847-supplementary.pdf]

Supplementary Material

Title: **Arbuscular mycorrhizal fungi and rhizobium improve nutrient uptake and microbial diversity relative to dryland site-specific soil conditions**

Rosalie B. Calderon[1*] and Sadikshya R. Dangi[1 *,†]

Rosalie,Calderon@usda,gov (Rosalie B. Calderon); Sadikshya.Dangi@usda.gov (Sadikshya R. Dangi)

[1]USDA ARS Northern Plains Agricultural Research Laboratory, Sidney, MT

Author for correspondence:

Rosalie B. Calderon

Rosalie.Calderon@usda.gov

Sadikshya R. Dangi

Sadikshya.Dangi@usda.gov

1500 North Central Ave., Sidney, Montana, USA

Tel: +1 406 433 9479, Fax: +1 406 433 5038

† Senior Author.

Contents

	Description	Page
Tables		
Table S1	Monthly mean temperature and precipitation from May to August during 2022 growing seasons at the two-dryland sites in Froid (DFS 1) and Sidney, Montana (DFS 2).	4
Table S2	Characterization of the soil physicochemical analysis of the two contrasting dryland sites	5
Table S3	Field management practices and chemical applications.	6
Table S4	Microbial dependency (%) data showing the microbial inoculants' contribution to aboveground biomass, grain yield and overall plant growth at two dryland sites.	7

Table S5	Pea nutrient requirement and baseline soil physicochemical analysis of the two contrasting dryland sites	8
Table S6	Effect of microbial inoculations on plant biomass and grain nutrient content (% by mass) at two dryland sites.	9
Table S7	Effect of microbial inoculants on carbon sequestered/stored in the harvested plant biomass and grains at two dryland sites.	10
Table S8	Effect of microbial inoculants on plant N and P nutrient uptake at two dryland sites.	11
Table S9	Effect of microbial inoculation on residual NPK (kg/ha) after pea cropping.	12
Table S10	Alpha and beta diversity of pea rhizosphere soils as influenced by microbial inoculants at two dryland conditions, MT 2022.	13
Table S11	Influence of microbial inoculants at two dryland sites on the most abundant (relative sequences abundance > 1% of all bacterial sequences) bacterial taxonomic group level phylum, order and genera on pea rhizosphere soil.	14
Table S12	Influence of microbial inoculants at two dryland sites on the most abundant (relative sequences abundance > 1% of all fungal sequences) fungal taxonomic group level phylum, order and genera on pea rhizosphere soil.	17
Table S13	Bacterial taxonomic difference between inoculated microbial communities and the control obtained from heat tree analysis, using the median abundance and non-parametric Wilcoxon Rank Sum test [56]	20
Table S14	Fungal taxonomic difference between inoculated microbial communities and the control obtained from heat tree analysis, using the median abundance and non-parametric Wilcoxon Rank Sum test [56].	22
Table S15	Influence of the microbial inoculants on the relative abundance of potential gene category involved in plant nutrient uptake at two dryland sites. The functional profiles of bacterial communities were predicted based on the 16S rRNA genes of retrieved bacterial taxa using Tax4Fun2 according to the KEGG Ortholog groups (KOs).	24

Table S16	The relative abundance of potential fungal traits in dryland condition. The ecological functional profiles of fungal communities were predicted based on the FungalTraits database.	25
Figures		
Figure S1	The field sites at (**a**) DFS 1(Froid) and (**b**) DFS 2 (Sidney) were managed under no-till practices. Each site was set up in 20,000 sq.ft. (USDA Soil Survey).	26
Figure S2	The yield components across dryland field sites. Two-way ANOVA analysis, means followed by different letters are significantly different according to Tukey's Test HSD at P <0.05 (n=400 plants).	26
Figure S3	Boxplots of the (**a**) plant stand, (**b**) nodulation rating scale using Yates et. al., 2017, and (**c**) plant biomass across dryland field sites. Two-way ANOVA analysis, means followed by different letters are significantly different according to LSD at P <0.05.	27
Figure S4	Influence of microbial inoculants on microbial species richness comparison among treatments and between sites on (**a, b**) bacterial, and (**c, d**) fungal communities. Boxplot and LS mean lines with common letter are not significantly different based on LSD tests at 0.05% probability level.	28

Table S1. Monthly mean temperature and precipitation from May to August during 2022 growing seasons at the two-dryland sites in Froid (DFS 1) and Sidney, Montana (DFS 2).

	Mean temperature (°C)		Mean precipitation (mm)	
Month	DFS1	DFS2	DFS1	DFS2
May	11.00	12.00	62.50	143.10
June	17.00	18.00	61.20	68.00
July	21.00	22.00	142.70	45.60
August	22.00	22.00	10.70	3.60
May -Aug	17.75	18.50	69.28	65.08

Weather data obtained from North Dakota Agricultural Weather Network National Oceanic and Atmospheric Administration, monthly and precipitation data (http://www.ndsu.nodak.edu/weather-data-monthly.html). The weather stations, Froid MT 5S and Sidney MT 1NW, were co-located at the research sites DFS 1 Froid and DFS 2 Sidney, MT. Websites accessed 8 November 2023.

Table S2 – Characterization of the soil physicochemical analysis of the two contrasting dryland sites.

Soil physico-chemical data	Dryland Site 1		Dryland Site 2	
Available water supply	0.17		0.18	
Soil Organic Matter (% LOI)	1.50	low	2.50	moderate
pH (1:1 pH rating)	4.70	strongly acidic	6.30	neutral
CEC/Sum of Cations me/100g	12.83		15.19	
Nutrients*†				
N	28.33	high	7.06	low
P	73.00	very high	44.75	high
K	214.50	very high	277.88	very high
S	10.85	medium	7.26	low
Ca	482.50	very high	1636.75	very high
Mg	140.63	very high	396.88	very high
Zn	0.59	medium	0.72	medium
Fe	69.16	very high	36.51	very high
Cu	0.68	very high	0.90	very high
Mn	102.13	very high	27.94	very high

*measured in parts per million (ppm).

† Soil fertility ratings for soil nutrients, Ward lab guide.

Table S3 – Field management practices and chemical applications.

Management practices	Category	Name	Active ingredient	Rate	Dryland site 1-Froid	Dryland site 2-Sidney
1. Tillage	NA	NA	NA	NA	No tillage	No tillage
2. Basal fertilization	Fertilizer	Ammonium phosphate	N, P	56 kg/ha	5/15/2022	5/17/2022
	Fertilizer	Muriate of Potash	K	45 kg/ha	5/15/2022	5/17/2022
3. Weed control	Herbicide	Basagran 5l	Bentazon	12.8 oz/acre (897g/ha)	6/27/2022	6/26/2022

Table S4 – Microbial dependency (%) data showing the microbial inoculants' contribution to aboveground biomass, grain yield and overall plant growth at two dryland sites.

Treatment	Biomass MD%	Grain MD%	Plant MD%
Microbial Inoculant (M)			
Control	0	0	0
AMF	-2.54	10.86	8.32
Rhizobium	1.51	2.70	4.21
AMF+ Rhizobium	1.16	10.65	11.81
Dryland Site (S)			
Site 1	-0.05	9.55	9.50
Site 2	0.11	2.56	2.67
M x S			
1_Control_DFS 1	0.00	0.00	0.00
2_AMF_DFS 1	-2.75	26.92	24.58
3_Rhizobium_DFS 1	3.68	2.08	5.76
4_AMF+Rhizobium_DFS 1	-1.53	9.19	7.66
5_Control_DFS 2	0.00	0.00	0.00
6_AMF_DFS 2	-2.75	-5.20	-7.94
7_Rhizobium_DFS 2	-1.53	3.32	2.66
8_AMF+Rhizobium_DFS 2	3.85	12.12	15.97
Significance			
M	ns‡	ns	ns
S	ns	ns	ns
M x S	ns	ns	ns

‡ Not significant.

Table S5– Pea nutrient requirement and baseline soil physicochemical analysis of the two contrasting dryland sites.

Soil physico-chemical data	Pea nutrient requirement[†]	Dryland Site 1		Dryland Site 2	
Available water supply		0.17		0.18	
Soil Organic Matter (% LOI)		1.50	low	2.50	moderate
pH (1:1 pH rating)	neutral	4.70	strongly acidic	6.30	Slightly neutral
Bulk density		1.60		1.60	
CEC/Sum of Cations me/100g		12.83		15.19	
Nutrients[†] (kg/ha)					
N	168	272	high	68	low
P	50	701	very high	430	very high
K	157	2,054	high	2,668	- high

[†] Pea nutrient requirement to produce average yield of 1,364 kg/ha

Table S6 – Effect of microbial inoculations on plant biomass and grain nutrient content (% by mass) at two dryland sites.

Treatment	Plant biomass			Pea grain			
	Carbon	Nitrogen	Protein	Carbon	Nitrogen	Phosphorus	Protein
Microbial Inoculant							
Control	42.80	1.43	8.92	41.76b[†]	4.27	0.43	26.66
AMF	42.70	1.47	9.18	42.04a	4.27	0.44	26.03
Rhizobium	42.90	1.49	9.32	41.91ab	4.28	0.43	26.75
AMF+ Rhizobium	42.50	1.50	9.35	42.03a	4.31	0.43	26.95
Dryland Site							
Site 1	42.60	2.16a	13.52a	42.54a	4.72a	0.40b	29.47a
Site 2	42.85	0.78b	4.86b	41.34b	3.80b	0.47a	23.73b
Significance							
M	ns[‡]	ns	ns	*	ns	ns	ns
S	**	**	**	**	**	**	**
M x S	ns	ns	ns	**	ns	ns	ns

* Significant $P \leq 0.05$.

**Significant $P \leq 0.001$.

[†] Means followed by different lowercase letter within a column are significantly different at $P \leq 0.05$ and $P \leq 0.001$.

[‡] Not significant.

Table S7 – Effect of microbial inoculants on carbon sequestered/stored in the harvested plant biomass and grains at two dryland sites.

Treatment	Biomass			Grain			Total C sequestered (kg/ha)
	yield (kg/ha)	C concentration	C content (kg/ha)	yield (kg/ha)	C concentration	C content (kg/ha)	
Microbial Inoculant							
Control	3146.89	0.4280	1346.43	1287.48	0.417615b[†]	536.69	1883.13
AMF	3004.30	0.4270	1282.19	1300.15	0.420349a	544.15	1826.34
Rhizobium	3117.00	0.4290	1337.09	1288.03	0.419117ab	536.40	1873.48
AMF+ Rhizobium	3154.65	0.4246	1340.46	1388.12	0.420333a	578.66	1919.12
Dryland Site							
Site 1	2916.21b[†]	0.4256	1241.48b	834.55b	0.4253a	355.06b	1596.54b
Site 2	3295.21a	0.4284	1411.60a	1797.35a	0.4133b	742.89a	2154.49a
Significance							
M	ns[‡]	ns	ns	ns	*	ns	ns
S	*	ns	*	**	**	**	**
M x S	ns	ns	ns	*	ns	*	ns

* Significant P≤0.05.

**Significant P≤0.001.

[†] Means followed by different lowercase letter within a column are significantly different at $P \leq 0.05$ and P≤0.001.

[‡] Not significant.

Table S8 – Effect of microbial inoculants on plant N and P nutrient uptake at two dryland sites.

Treatment	N uptake in biomass	N uptake in grains	N uptake whole plant	P uptake in grains
Microbial Inoculant				
Control	43.74	53.22	96.95	5.93946
AMF	43.29	52.55	95.85	6.19661
Rhizobium	46.12	52.78	98.90	5.65876
AMF+ Rhizobium	45.21	57.00	102.21	6.17326
Dryland Site				
Site 1	63.67a†	39.42b	102.79	3.37656b
Site 2	25.81b	68.36a	94.17	8.60748a
Significance				
M	ns‡	ns	ns	ns
S	**	**	ns	**
M x S	ns	ns	ns	ns

**Significant P≤0.001.

† Means followed by different lowercase letter within a column are significantly different at $P \leq 0.05$ and P≤0.001.

‡ Not significant.

Table S9 –Effect of microbial inoculation on soil organic residual NPK (kg/ha) after pea cropping.

Soil nutrient residual	N	P	K
Microbial inoculants (M)			
Control	40.90	130.80	1,358.40
AMF	58.44	124.80	1,334.40
Rhizobium	46.56	96.00	1,194.00
AMF+ Rhizobium	41.28	120.00	1,327.00
Sites (S)			
Dryland site 1	68.16a[†]	162.60a	1,204.40b
Dryland site 2	25.44b	73.20b	1,401.60a
Significance			
M	ns[‡]	ns	ns
S	**	**	*
M x S	ns	ns	ns

* Significant P≤0.05 **Significant P≤0.001 [‡] Not significant

[†] Means followed by different lowercase letter within a column are significantly different at $P \leq 0.05$ and P≤0.001. Table S10. Alpha and beta diversity of pea rhizosphere soils as influenced by microbial inoculants at two dryland conditions, MT 2022.

Diversity metrics	Microbial Inoculants (M)	Dryland Sites (S)	M X S
Bacterial and archaeal community (16S rRNA)			
† Alpha diversity			
Observed ASVs	0.029	0.143	0.008
Shannon index	0.041	0.354	0.017
‡Beta diversity	0.637 / R^2=0.065	0.001** / R^2=0.314	0.001** / R^2=0.436
Fungal community (ITS)			
† Alpha diversity			
Observed ASVs	0.409	1.02e-07**	0.003
Shannon index	0.852	0.00004**	0.043*
‡Beta diversity	0.998/ R^2=0.027*	0.001* / R^2=0.484	0.001** / R^2=0.551

* Significant P≤0.05.

** Significant P≤0.001.

† Alpha diversity metrics, a qualitative measure of microbial richness using observed species richness and Shannon diversity index

‡ Beta diversity metrics, a quantitative measure of community dissimilarity using Bray Curtis Index (statistical method: permutational analysis of variance (PERMANOVA).

Table S11 - Influence of microbial inoculants at two dryland sites on the most abundant (relative sequences abundance > 1% of all bacterial sequences) bacterial taxa on pea rhizosphere soil.

Taxonomic group	Microbial Inoculants (M)				Dryland Site (S)		Significance P value		
**Phylum**	Control	AMF	Rhizobium	AMF+ Rhizobium	Site 1	Site 2	M	S	M*S
Actinobacteria	0.35964b†	0.32322b	0.43008a	0.35661b	0.35426	0.38052	*	ns‡	ns
Proteobacteria	0.25526	0.22502	0.24957	0.28519	0.25726	0.25026	ns	ns	ns
Acidobacteria	0.13851	0.22504	0.13422	0.1437	0.15621	0.16453	ns	ns	ns
Chloroflexi	0.07418	0.04076	0.06432	0.06324	0.06401	0.05724	ns	ns	ns
Bacteroidetes	0.07443	0.04453	0.04102	0.05335	0.04092b	0.06574a	ns	*	ns
WPS2	0.03683	0.06741	0.03226	0.03739	0.08694a	-2.10E-17b	ns	**	ns
Firmicutes	0.02311	0.03897	0.02457	0.02579	0.00792b	0.0483a	ns	**	ns
Thaumarchaeota	0.01374	0.01959	0.01177	0.0145	0.00963	0.02017	ns	ns	ns
Nitrospirae	0.00886	0.0081	0.0016	0.00555	-4.30E-18b	0.01206a	ns	**	ns
Planctomycetes	0.00857	0.00204	0.00594	0.00685	0.0105a	0.0012b	ns	**	ns
**Order**									
Propionibacteriales	0.11409	0.10717	0.13561	0.13655	0.05045b	0.19626a	ns‡	*	ns
Solibacterales	0.06859	0.10651	0.08728	0.09069	0.09694	0.07959	ns	ns	ns
Betaproteobacteriales	0.09407	0.06969	0.096	0.0898	0.07107	0.10371	ns	ns	ns
Micrococcales	0.06667	0.07355	0.08214	0.07485	0.08638	0.06223	ns	ns	ns
Solirubrobacterales	0.06814	0.05818	0.08691	0.05131	0.08252	0.04975	ns	ns	ns
Rhizobiales	0.05325	0.07277	0.06797	0.07511	0.04594b	0.0746a	ns	*	ns
Acetobacterales	0.05777	0.05516	0.0584	0.05663	0.1029a	0.01108b	ns	**	ns

Order	Control	AMF	Rhizobium	AMF+ Rhizobium	Site 1	Site 2	M	S	M*S
Frankiales	0.060a	0.0284c	0.07489a	0.04422ab	0.03272b	0.07112a	*	**	ns
Chitinophagales	0.07443	0.05335	0.04453	0.04102	0.04092b	0.06574a	ns	*	ns
Uncultured bacterium	0.03683	0.06741	0.03226	0.03739	0.08694a	-2.10E-17b	ns	**	ns
Elsterales	0.0368b	0.0274b	0.02551b	0.06897a	0.01848b	0.06087a	*	*	*
Acidobacteriales	0.0235b	0.0613a	0.01577b	0.02857b	0.05927a	0.00529b	*	**	ns
Thermomicrobiales	0.0371	0.02738	0.02721	0.02945	0.00333b	0.05724a	ns	**	ns
Bacillales	0.02311	0.03897	0.02457	0.02579	0.00792b	0.0483a	ns	**	ns
Gaiellales	0.0291	0.02535	0.03131	0.0264	0.05608a	-3.50E-18b	ns	**	ns
**Genus**									
uncultured bacterium	0.14048	0.13688	0.12272	0.17982	0.2202a	0.0698b	ns‡	*	ns
Nocardioides	0.10011	0.08104	0.10245	0.11032	0.04745b	0.14952a	ns	*	ns
Bryobacter	0.06859	0.10651	0.08728	0.09069	0.09694	0.07959	ns	ns	ns
uncultured	0.0919	0.08109	0.04496	0.06138	0.08143	0.05823	ns	ns	*
Pseudarthrobacter	0.04773	0.05247	0.06399	0.0477	0.04844	0.05751	ns	ns	ns
Blastococcus	0.0602	0.02839	0.07489	0.04422	0.03272b	0.0711a	*	**	ns
Conexibacter	0.04512	0.03362	0.05427	0.03204	0.08252a	2.08E-17b	ns	**	ns
Acidiphilium	0.03438	0.03739	0.04175	0.03859	0.06498a	0.0111b	ns	**	ns
Bradyrhizobium	0.0237	0.04127	0.03026	0.02984	0.02436	0.03817	ns	ns	ns
uncultured Acidobacteria	0.02606	0.06366	0.0149	0.02001	0.01939	0.04293	ns	ns	ns
uncultured Chloroflexi	0.04166	0.02301	0.03142	0.02667	0.0126	0.04878	ns	ns	ns

Supplementary Material

**Genus**	Control	AMF	Rhizobium	AMF+ Rhizobium	Site 1	Site 2	M	S	M*S
uncultured bacterium	0.14048	0.13688	0.12272	0.17982	0.2202a	0.0698b	ns‡	*	ns
Nocardioides	0.10011	0.08104	0.10245	0.11032	0.04745b	0.14952a	ns	*	ns
Bryobacter	0.06859	0.10651	0.08728	0.09069	0.09694	0.07959	ns	ns	ns
uncultured	0.0919	0.08109	0.04496	0.06138	0.08143	0.05823	ns	ns	*
Pseudarthrobacter	0.04773	0.05247	0.06399	0.0477	0.04844	0.05751	ns	ns	ns
Blastococcus	0.0602	0.02839	0.07489	0.04422	0.03272b	0.0711a	*	**	ns
Conexibacter	0.04512	0.03362	0.05427	0.03204	0.08252a	2.08E-17b	ns	**	ns
Acidiphilium	0.03438	0.03739	0.04175	0.03859	0.06498a	0.0111b	ns	**	ns
Bradyrhizobium	0.0237	0.04127	0.03026	0.02984	0.02436	0.03817	ns	ns	ns
uncultured Acidobacteria	0.02606	0.06366	0.0149	0.02001	0.01939	0.04293	ns	ns	ns
uncultured Chloroflexi	0.04166	0.02301	0.03142	0.02667	0.0126	0.04878	ns	ns	ns
Bacillus	0.02311	0.03897	0.02457	0.02579	0.00792b	0.0483a	ns	**	ns
Solirubrobacter	0.02303	0.02457	0.03264	0.01927	1.73E-17b	0.0498a	ns	*	ns
Massilia	0.03641	0.00797	0.02941	0.02226	0.02306	0.02496	ns	ns	ns
Rhizobacter	0.0203	0.0181	0.02523	0.02187	0.00666b	0.0361a	ns	*	ns
RB41	0.02639	0.01818	0.0217	0.01775	-3.50E-18b	0.0420a	ns	*	ns
Microlunatus	0.00559	0.02204	0.02698	0.01761	-6.90E-18b	0.0361a	ns	**	ns
Nitrosospira	0.0156	0.01188	0.01966	0.01818	0.00318b	0.0295a	ns	**	ns
Allorhizobium-Neorhizobium-Pararhizobium-Rhizobium	0.0033	0.0000	0.0337	0.00470	0.0075b	0.0341a	ns	**	ns

* Significant P≤0.05; **Significant P≤0.001; † Means followed by different lowercase letter within a column are significantly different at $P \leq 0.05$; and ‡ Not significant.

Table S12 - Influence of microbial inoculants at two dryland sites on the most abundant (relative sequences abundance) fungal taxonomic group level phylum, order and genera on pea rhizosphere soil.

Taxonomic group	Microbial Inoculants (M)				Dryland Site (S)		Significance P value		
**Phylum**	Control	AMF	Rhizobium	AMF+ Rhizobium	Site 1	Site 2	M	S	M*S
Ascomycota	0.33735	0.32257	0.32465	0.35048	0.71759	0.61748	ns‡	ns	ns
Basidiomycota	0.08992	0.08083	0.09843	0.07631	0.18591	0.15959	ns	ns	ns
Mortierellomycota	0.03459	0.04533	0.04541	0.03270	0.04843b†	0.10963a	ns	*	ns
Chytridiomycota	0.01901	0.02219	0.01556	0.02552	0.02659b	0.05569a	ns	*	ns
unidentified	0.02737	0.01206	0.01528	0.01444	0.01616	0.01344	ns	ns	ns
Not_Assigned	0.01628	0.0264	0.01423	0.01305	0.01616b	0.02344a	ns	*	ns
Mucoromycota	0.00137	0.00204	0.00067	0.00088	0.00492a	5.2E-05b	ns	*	ns
Glomeromycota	0.00124	0.00053	0.00089	0.00086	0.00020b	0.00338a	ns	*	ns
Rozellomycota	0.00019	0.00011	0.00010	0.00017	0.00020	0.00037	ns	ns	ns
Zoopagomycota	0	8.27E-06	0	2.06E-05	8.36E-06	2.06E-05	ns	ns	ns
**Order**									
Hypocreales	0.16429	0.15973	0.16468	0.17425	0.15646	0.17502	ns‡	ns	ns
Eurotiales	0.16051	0.12354	0.13151	0.13789	0.21502a	0.06171b	ns	**	ns
Unidentified	0.09202	0.13852	0.13361	0.14573	0.11849	0.13645	ns	ns	ns
Mortierellales	0.08086	0.09242	0.06367	0.07245	0.03782b	0.11687a	ns	*	ns
Pleosporales	0.08641	0.07649	0.09024	0.07161	0.04552b	0.11686a	ns	**	ns
Filobasidiales	0.09841	0.06129	0.06470	0.06520	0.09796a	0.04684b	ns	**	ns
Thelebolales	0.05367	0.07850	0.07694	0.05500	0.08226	0.04980	ns	ns	ns

**Order**	Control	AMF	Rhizobium	AMF+ Rhizobium	Site 1	Site 2	M	S	M*S
Sordariales	0.04105	0.05648	0.05636	0.05399	0.07766a	0.02628b	ns	**	ns
Not Assigned	0.04251	0.04473	0.06196	0.04136	0.02641b	0.06887a	ns	*	ns
Coniochaetales	0.02919	0.03191	0.01882	0.03258	0.00223b	0.05402a	ns	**	ns
Helotiales	0.03294	0.02634	0.02111	0.03160	0.00626b	0.04974a	ns	*	ns
Chaetothyriales	0.02475	0.02865	0.02596	0.02624	0.02338	0.02943	ns	ns	ns
Holtermanniales	0.01958	0.01718	0.01148	0.01103	0.02927a	0.00036b	ns	**	ns
Tremellales	0.01056	0.01152	0.01578	0.01574	0.02622a	0.00059b	ns	**	ns
Glomerales	0.00869	0.00471	0.00983	0.01079	0.00112b	0.01588a	ns	**	ns
**Genus**									
unidentified	0.13734	0.15375	0.13567	0.17872	0.11384b	0.75561a	ns‡	*	ns
Mortierella	0.06578	0.08698	0.08737	0.06152	0.04273b	0.43240a	ns	*	ns
Hamigera	0.10169	0.06048	0.07182	0.0863	0.16017a	0b	ns	**	ns
Not Assigned	0.08824	0.11895	0.09874	0.0864	0.058639b	0.55012a	ns	*	ns
Pseudogymnoascus	0.05140	0.07147	0.07968	0.05151	0.08181b	0.18099a	ns	ns	ns
Naganishia	0.05079	0.03482	0.05396	0.03430	0.07527b	0.04666a	ns	**	ns
Penicillium	0.05516	0.0514	0.04861	0.04925	0.04831b	0.21567a	ns	ns	ns
Gibberella	0.03919	0.03173	0.04926	0.03899	0.03511b	0.17791a	ns	*	ns
Fusicolla	0.03233	0.03817	0.03411	0.03813	0.0488b	0.09024a	ns	**	ns
Clonostachys	0.02787	0.02586	0.016552	0.025869	0.03126b	0.06724a	ns	*	ns

**Genus**	Control	AMF	Rhizobium	AMF+ Rhizobium	Site 1	Site 2	M	S	M*S
Fusarium	0.02265	0.01882	0.023622	0.02975	0.00750b	0.15967a	ns	**	ns
Chrysanthotrichum	0.01231	0.02952	0.018671	0.03158	0.04604a	0b	ns	**	ns
Solicoccozyma	0.02932	0.01477	0.015267	0.02181	0.01084b	0.11898a	ns	*	ns
Trichoderma	0.01584	0.02104	0.009673	0.01601	0.0205b	0.04309a	ns	*	ns
Knufia	0.01414	0.01548	0.014616	0.01502	0.01234b	0.06918a	ns	*	ns
Coniochaeta	0.01114	0.01642	0.010094	0.0211	0.0020b	0.10944a	ns	**	ns
Holtermanniella	0.01661	0.01519	0.01392	0.01041	0.0277a	0.0016b	ns	**	ns
Neosetophoma	0.01910	0.01102	0.00985	0.00719	0.00313b	0.08180a	ns	**	ns
Sclerostagonospora	0.01299	0.01107	0.011306	0.01166	0.00796b	0.06221a	ns	*	ns
Saitozyma	0.00927	0.00924	0.018171	0.01025	0.02318a	0.0012b	ns	**	ns
Glomus	0.00034	1.09E-05	0	0.00026	0b	0.00122a	ns	**	ns

* Significant P≤0.05.

**Significant P≤0.001.

† Means followed by different lowercase letter within a column are significantly different at *P* ≤ 0.05.

‡ Not significant

Table S13 – Bacterial and archaeal taxonomic difference between inoculated microbial communities and the control obtained from heat tree analysis, using the median abundance and non-parametric Wilcoxon Rank Sum test [56].

tax_rank	tax_name	log2_median_ ratio	median_diff	mean_diff	wilcox_ p_value
**Dryland Site 1**					
**AMF vs Control**					
o__D_3	Elsterales	Inf	-0.02564	-0.02667	0.00729
o__D_3	Xanthomonadales	Inf	-0.02564	-0.03795	0.00729
f__D_4	uncultured	Inf	-0.02564	-0.02667	0.00729
f__D_4	Rhodanobacteraceae	-1.442820656	-0.02564	-0.03795	0.00729
g__D_5	uncultured_bacterium	Inf	-0.02564	-0.02667	0.00729
s__D_6	uncultured_bacterium	Inf	-0.02564	-0.02667	0.00729
c__D_2	Chloroflexia	Inf	-0.01026	-0.01231	0.00729
p__D_1	Chloroflexi	Inf	-0.09231	-0.06256	0.020008
s__D_6	uncultured_bacterium	Inf	-0.01026	-0.01231	0.024251
o__D_3	Elev_1554	Inf	-0.00513	-0.00718	0.024808
f__D_4	uncultured_Chloroflexi_bacterium	Inf	-0.03077	-0.02154	0.024808
f__D_4	uncultured_bacterium	Inf	-0.00513	-0.00718	0.024808
g__D_5	uncultured_Chloroflexi_bacterium	Inf	-0.03077	-0.02154	0.024808
g__D_5	uncultured_bacterium	Inf	-0.00513	-0.00718	0.024808
s__D_6	uncultured_Chloroflexi_bacterium	Inf	-0.03077	-0.02154	0.024808
s__D_6	uncultured_bacterium	Inf	-0.00513	-0.00718	0.024808
g__D_5	Chujaibacter	Inf	-0.02051	-0.02359	0.02537
g__D_5	Segetibacter	Inf	-0.01026	-0.01026	0.02537
p__D_1	Acidobacteria	Inf	0.082051	0.164103	0.055556
c__D_2	Acidobacteriia	Inf	0.082051	0.164103	0.055556
c__D_2	Ktedonobacteria	Inf	-0.07692	-0.05026	0.057008
g__D_5	Rhizobacter	Inf	-0.00513	-0.00513	0.070701
**Rhizobium vs Control**					
s__D_6	uncultured_bacterium	0.736966	0.030769	0.049231	0.015651
g	Ambiguous_taxa	1.321928	0.015385	0.020513	0.089686
s	Ambiguous_taxa	1.321928	0.015385	0.020513	0.089686
s__D_6	uncultured_Conexibacteraceae_bacterium	Inf	-0.04103	-0.02462	0.105998
p__D_1	Chloroflexi	-0.58496	-0.03077	-0.01641	0.141238
**AMF+Rhizobium**					
s__D_6	uncultured_bacterium	Inf	-0.03077	-0.02359	0.025921

tax_rank	tax_name	log2_median_ratio	median_diff	mean_diff	wilcox_p_value
c__D_2	Ktedonobacteria	-0.58496	-0.02564	-0.02974	0.058553
p__D_1	Chloroflexi	-0.46949	-0.02564	-0.02667	0.059327
s__D_6	uncultured_bacterium	1.321928	0.015385	0.013333	0.088683
s__D_6	uncultured_bacterium	0.415037	0.015385	0.033846	0.093693
f__D_4	Burkholderiaceae	0.485427	0.020513	0.027692	0.095238
**Dryland Site 2**					
**AMF vs Control**					
p__D_1	Bacteroidetes	-1.28951	-0.06667	-0.0759	0.021177
c__D_2	Bacteroidia	-1.28951	-0.06667	-0.0759	0.021177
o__D_3	Chitinophagales	-1.28951	-0.06667	-0.0759	0.021177
f__D_4	Chitinophagaceae	-1.28951	-0.06667	-0.0759	0.021177
g__D_5	uncultured	-1.28951	-0.06667	-0.07692	0.021177
o__D_3	Propionibacteriales	0.440573	0.051282	0.051282	0.074913
f__D_4	Propionibacteriaceae	Inf	0.051282	0.030769	0.156337
**Rhizobium vs Control**					
p__D_1	Actinobacteria	0.55849	0.133333	0.154872	0.007937
c__D_2	Actinobacteria	0.55849	0.133333	0.154872	0.007937
o__D_3	Micrococcales	0.584963	0.025641	0.036923	0.011412
f__D_4	Micrococcaceae	0.584963	0.025641	0.043077	0.011667
g__D_5	Pseudarthrobacter	0.584963	0.025641	0.043077	0.011667
s__D_6	Pseudarthrobacter_polychromogenes	0.584963	0.025641	0.043077	0.011667
o__D_3	Propionibacteriales	0.550197	0.066667	0.094359	0.055556
**AMF+Rhizobium**					
o__D_3	Elsterales	1.307431499	0.06323187	0.071085769	**0.036145**
f__D_4	uncultured	1.307431499	0.06323187	0.071085769	**0.036145**
g__D_5	uncultured_bacterium	1.307431499	0.06323187	0.071085769	**0.036145**
s__D_6	uncultured_bacterium	1.307431499	0.06323187	0.071085769	**0.036145**
p__D_1	Bacteroidetes	-0.494478566	-0.02973174	-0.041845041	0.055556
c__D_2	Bacteroidia	-0.494478566	-0.02973174	-0.041845041	0.055556
o__D_3	Chitinophagales	-0.494478566	-0.02973174	-0.041845041	0.055556
f__D_4	Chitinophagaceae	-0.494478566	-0.02973174	-0.041845041	0.055556

Table S14- Fungal taxonomic difference between inoculated microbial communities and the control obtained from heat tree analysis, using the median abundance and non-parametric Wilcoxon Rank Sum test [56].

tax_ rank	tax_name	log2_median _ratio	median_ diff	mean_diff	wilcox_ p_value
**Dryland Site 1**					
**AMF vs Control**					
o_o	unidentified	0.963163	0.036323	0.043266	0.007937
f_f	unidentified	0.963163	0.036323	0.043266	0.007937
g_g	unidentified	0.963163	0.036323	0.043266	0.007937
s	unidentified	0.963163	0.036323	0.043266	0.007937
o_o	Dothideales	Inf	0.001286	0.001093	0.031141
f_f	Dothideales_fam_Incertae_sedis	Inf	0.001286	0.001093	0.031141
g_g	Selenophoma	Inf	0.001286	0.001093	0.031141
s	Selenophoma_mahoniae	Inf	0.001286	0.001093	0.031141
o_o	Pleosporales	0.810339	0.016715	0.018515	0.031746
s	Keissleriella_poagena	Inf	0.005143	0.003729	0.034454
f_f	Lentitheciaceae	3	0.00675	0.006493	0.036145
g_g	Keissleriella	3	0.00675	0.006493	0.036145
g_g	Parastagonospora	2	0.002893	0.002893	0.036145
c_c	Sordariomycetes	0.598915	0.113468	0.106332	0.055556
**Rhizobium vs Control**					
o_o	Pleosporales	0.779401	0.015429	0.033044	0.015873
f_f	Phaeosphaeriaceae	1.681824	0.017036	0.018579	0.015971
s	Keissleriella_poagena	Inf	0.007393	0.007457	0.020008
s	Coniochaeta_discospora	Inf	0.001607	0.001543	0.024808
g_g	Chrysanthotrichum	1.577976	0.044037	0.03298	0.031746
s	Chrysanthotrichum_peruvianum	1.577976	0.044037	0.03298	0.031746
f_f	Lentitheciaceae	3.906891	0.0135	0.010158	0.036145
g_g	Keissleriella	3.906891	0.0135	0.010158	0.036145
g_g	Parastagonospora	2	0.003857	0.00405	0.046533
c_c	Dothideomycetes	0.63941	0.018644	0.034523	0.055556
**AMF+Rhizobium**					
o_o	Pleosporales	0.779401	0.015429	0.033044	0.015873
f_f	Phaeosphaeriaceae	1.681824	0.017036	0.018579	0.015971
s	Keissleriella_poagena	Inf	0.007393	0.007457	0.020008
s	Coniochaeta_discospora	Inf	0.001607	0.001543	0.024808
g_g	Chrysanthotrichum	1.577976	0.044037	0.03298	0.031746
s	Chrysanthotrichum_peruvianum	1.577976	0.044037	0.03298	0.031746
f_f	Lentitheciaceae	3.906891	0.0135	0.010158	0.036145

tax_ rank	tax_name	log2_median _ratio	median_ diff	mean_diff	wilcox_ p_value
g_g	Keissleriella	3.906891	0.0135	0.010158	0.036145
g_g	Parastagonospora	2	0.003857	0.00405	0.046533
c_c	Dothideomycetes	0.63941	0.018644	0.034523	0.055556
**Dryland Site 2**					
**AMF vs Control**					
s	Exophiala_radicis	Inf	-0.00064	-0.00154	0.018119
s	Naganishia_diffluens	Inf	-0.00161	-0.00231	0.044171
g_g	Myrothecium	Inf	-0.00064	-0.00058	0.072006
**Rhizobium vs Control**					
f__	Bulleribasidiaceae	Inf	-0.00032	-0.00039	0.024251
g_g	Dioszegia	Inf	-0.00032	-0.00039	0.024251
s	unidentified	Inf	-0.00032	-0.00039	0.024251
g__	Articulospora	Inf	0.000321	0.0009	0.024808
s	Articulospora_proliferata	Inf	0.000321	0.0009	0.024808
c_c	Rhizophlyctidomycetes	Inf	-0.00161	-0.00154	0.072006
**AMF+Rhizobium**					
c_c	Sordariomycetes	0.164795	0.04018	0.071167	0.007937
s	Exophiala_lacus	Inf	0.001286	0.000964	0.024808
s	Mortierella_sarnyensis	0.658963	0.007072	0.010929	0.027803
f_f	Phaeosphaeriaceae	-1.37137	-0.05561	-0.03967	0.031746
o_o	Tremellales	Inf	-0.00064	-0.0009	0.039318
s	Mortierella_fimbricystis	Inf	0.00225	0.002314	0.044909
c_c	Dothideomycetes	-0.7929	-0.06075	-0.06519	0.055556
o_o	Pleosporales	-0.73571	-0.04918	-0.05773	0.055556

Table S15- Influence of the microbial inoculants on the relative abundance of potential gene category involved in plant nutrient uptake at two dryland sites. The functional profiles of bacterial communities were predicted based on the 16S rRNA genes of retrieved bacterial taxa using Tax4Fun2 according to the KEGG Ortholog groups (KOs).

Potential gene category	Microbial Inoculants (M)				Dryland Site (S)		Significance P value		
	Control	AMF	Rhizobium	AMF+ Rhizobium	Site 1	Site 2	M	S	M*S
Carbon fixation	0.010655	0.011127	0.010986	0.011175	0.00995b[†]	0.0120a	ns[‡]	**	ns
Nitrogen metabolism	0.003088	0.00293	0.002929	0.002996	0.00248b	0.0035a	ns	**	ns
Complete nitrification	0.000532	0.000351	0.000457	0.000485	0.000204	0.00071	ns	**	ns
Nitrification	0.001896	0.001955	0.00188	0.001892	0.00179b	0.002023a	ns	*	ns
Assimilatory nitrate reduction	0.000651	0.000602	0.000583	0.000609	0.00047b	0.0008a	ns	**	ns
Nitrogen fixation	9.10E-06	0.000023	9.70E-06	0.00001	1.35E-05	1.2E-05	ns	ns	ns
Phosphorus	0.013068	0.012239	0.012631	0.012698	0.012576	0.01274	ns	ns	ns
P transport	0.005302	0.005144	0.00524	0.005137	0.005135	0.00528	ns	ns	ns
P solubilization	0.005705	0.005145	0.005413	0.005594	0.005396	0.00553	ns	ns	ns
P starvation regulation	0.002061	0.00195	0.001978	0.001967	0.002045	0.00193	ns	ns	ns

* Significant P≤0.05.

**Significant P≤0.001.

[†] Means followed by different lowercase letter within a column are significantly different at $P \leq 0.05$.

[‡] Not significant

Table S16- The relative abundance of potential fungal traits in dryland condition. The ecological functional profiles of fungal communities were predicted based on the FungalTraits database.

Ecological fungal traits	Microbial Inoculants (M)				Dryland Site (S)		Significance P value		
	Control	AMF	Rhizobium	AMF+ Rhizobium	Site 1	Site 2	M	S	M*S
Saprotrophs	1850.5	1765.5	1803.2	1721.4	2086a†	1484.3b	ns‡	**	ns
Arbuscular_mycorrhizal	4.8	0.7	3.7	2.2	-4.0E-15b	5.7a	ns	**	ns
Animal/insect_parasite	30.7	31.3	20.4	23	10.8b	41.9a	ns	*	ns
Mycoparasite	172.1	210.6	172.1	183.7	246.65a	122.6b	ns	**	ns
Plant_pathogen	361.7	288.3	384.7	372.7	250.45b	453.3a	ns	**	ns
Others	474.5	523.2	499.5	619.6	416.25b	642.2a	ns	**	ns

* Significant P≤0.05.

**Significant P≤0.001.

† Means followed by different lowercase letter within a column are significantly different at $P \leq 0.05$ and P≤0.001.

‡ Not significant

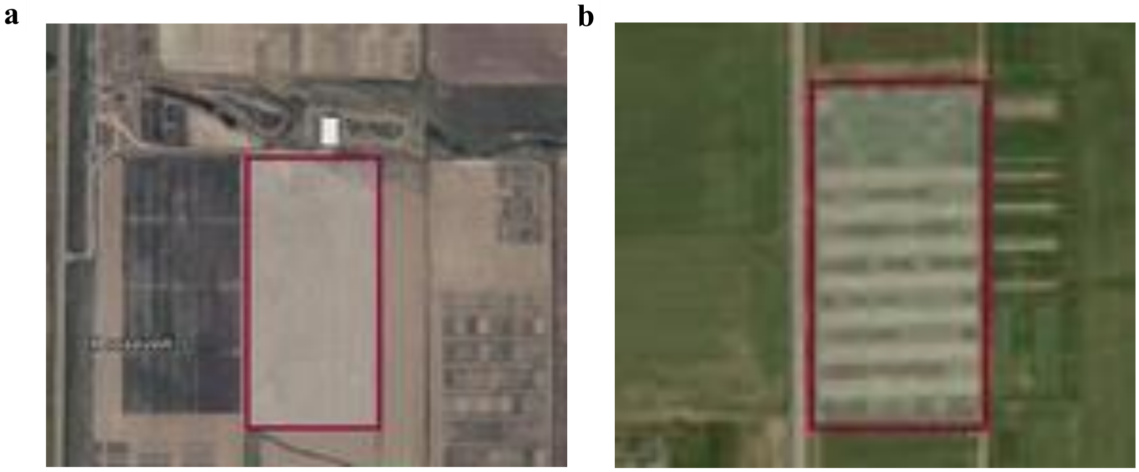

Supplementary Figure S1. The field sites at DFS 1(Froid) (**a**) and DFS 2 (Sidney) (**b**) were managed under no-till practices. Each site was set up in 20,000 sq.ft. with four treatments: Control, AMF, Rhizobium, and dual AMF and rhizobial inoculants in a randomized complete block design (RCBD) with 5 replications.

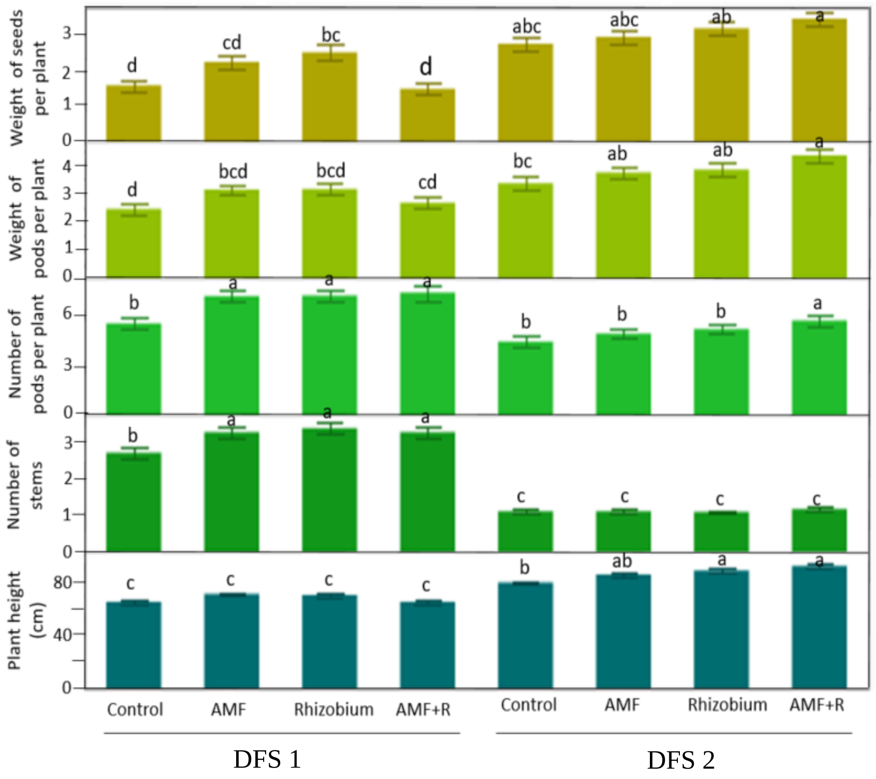

Supplementary Figure S2. The yield components across dryland field sites. Two-way ANOVA analysis, means followed by different letters are significantly different according to Tukey's Test HSD at P <0.05 (n=400 plants).

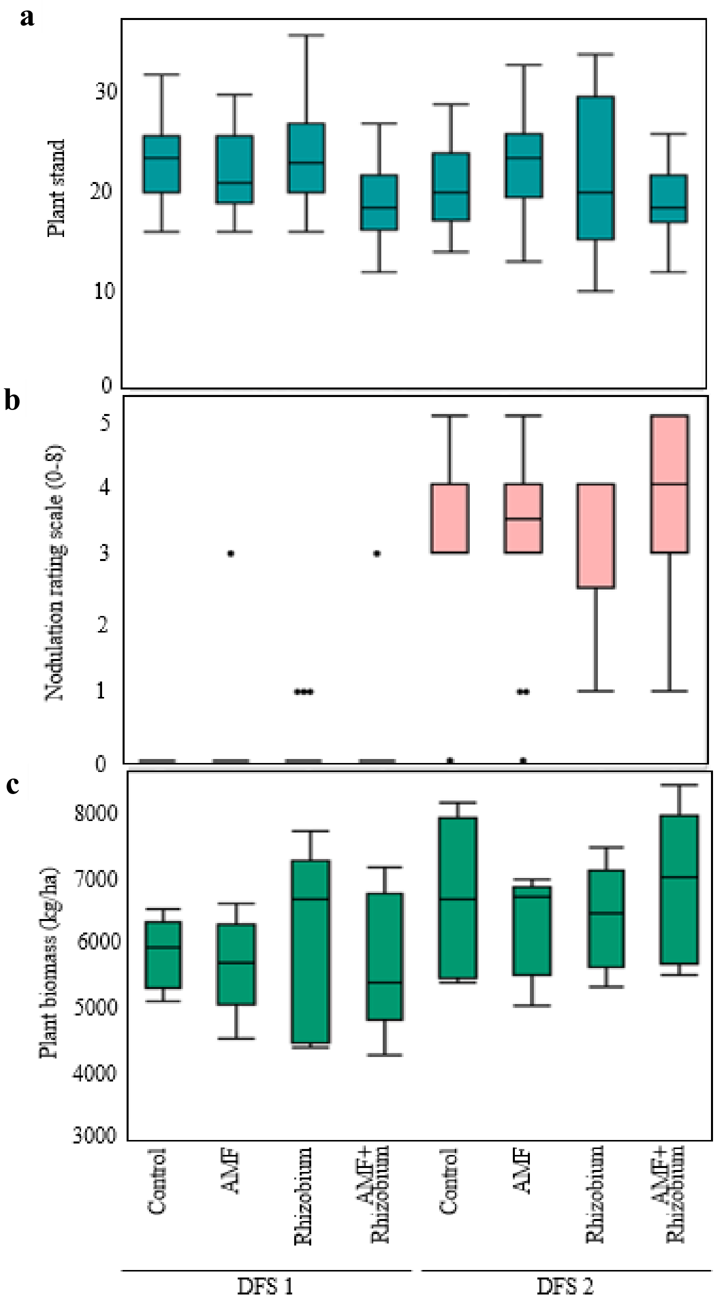

Supplementary Figure S3. Boxplots of the (**a**) plant stand, (**b**) nodulation rating scale using Yates et al. (2016) [84], and (**c**) plant biomass across dryland field sites. Two-way ANOVA analysis, means followed by different letters are significantly different according to LSD at P <0.05.

Yates, R J, R Abaidoo, and J G Howieson. 2016. "Field Experiments with Rhizobia." In . Australian Centre for International Agricultural Research.

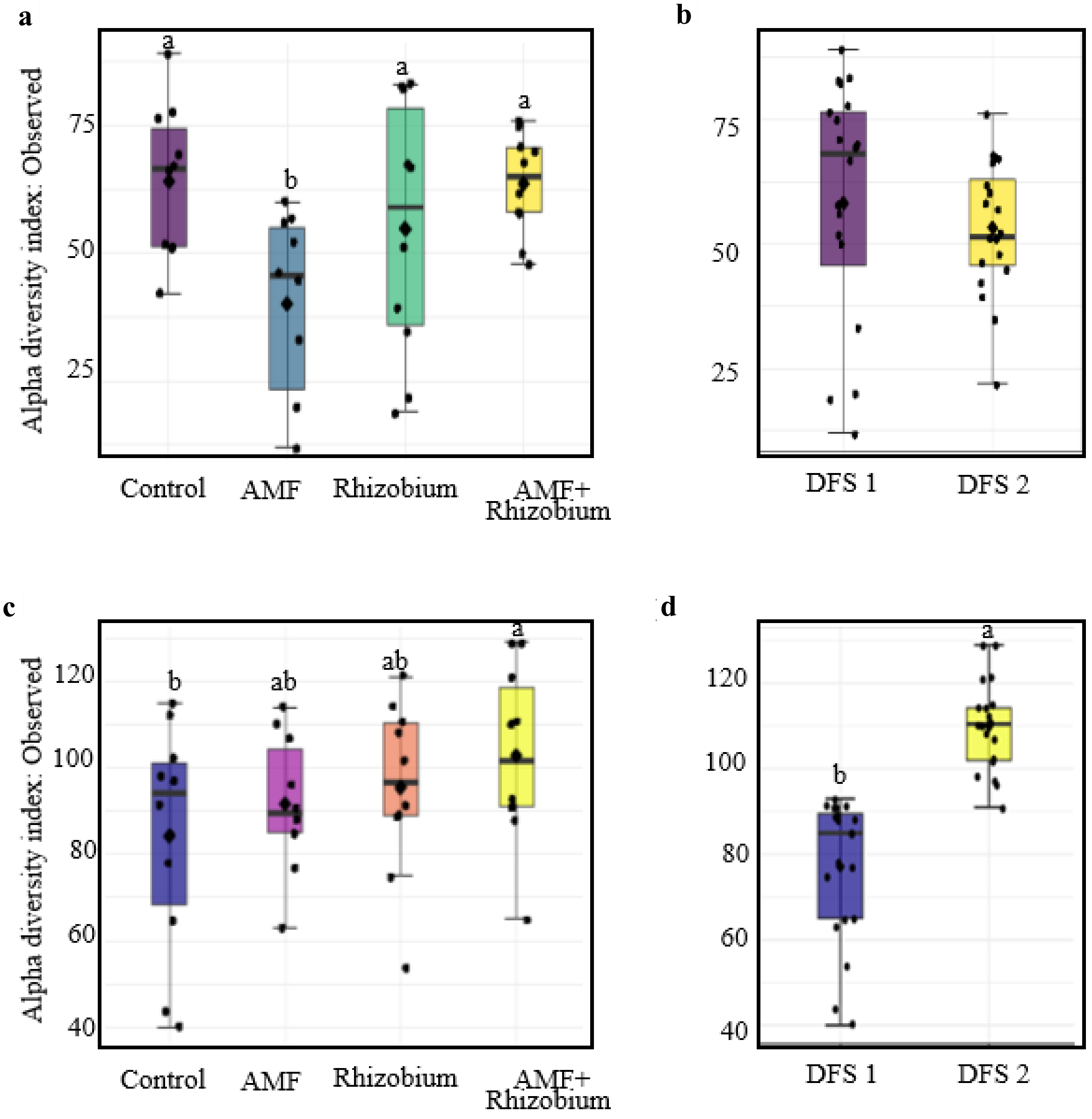

Supplementary Figure S4. Influence of microbial inoculants on microbial species richness comparison among treatments and between sites on (**a, b**) bacterial, and (**c, d**) fungal communities. Boxplot and LS mean lines with common letter are not significantly different based on LSD tests at 0.05% probability level.
